# The Composite of Bone Marrow Concentrate and PRP as an Alternative to Autologous Bone Grafting

**DOI:** 10.1371/journal.pone.0100143

**Published:** 2014-06-20

**Authors:** Mohssen Hakimi, Jan-Peter Grassmann, Marcel Betsch, Johannes Schneppendahl, Sebastian Gehrmann, Ahmad-Reza Hakimi, Patric Kröpil, Martin Sager, Monika Herten, Michael Wild, Joachim Windolf, Pascal Jungbluth

**Affiliations:** 1 Department of Trauma and Handsurgery, Heinrich Heine University Hospital Duesseldorf, Duesseldorf, Germany; 2 Animal Research Institute, Heinrich Heine University Hospital Duesseldorf, Duesseldorf, Germany; 3 Department of Oral Surgery, Heinrich Heine University Hospital Duesseldorf, Duesseldorf, Germany; 4 Department of Orthopaedics, Heinrich Heine University Hospital Duesseldorf, Duesseldorf, Germany; 5 Department of Diagnostic and Interventional Radiology, Heinrich Heine University Hospital Duesseldorf, Duesseldorf, Germany; INSERM U1059/LBTO, Université Jean Monnet, France

## Abstract

One possible alternative to the application of autologous bone grafts represents the use of autologous bone marrow concentrate (BMC). The purpose of our study was to evaluate the potency of autologous platelet-rich plasma (PRP) in combination with BMC. In 32 mini-pigs a metaphyseal critical-size defect was surgically created at the proximal tibia. The animals were allocated to four treatment groups of eight animals each (1. BMC+CPG group, 2. BMC+CPG+PRP group, 3. autograft group, 4. CPG group). In the BMC+CPG group the defect was filled with autologous BMC in combination with calcium phosphate granules (CPG), whereas in the BMC+CPG+PRP group the defect was filled with the composite of autologous BMC, CPG and autologous PRP. In the autograft group the defect was filled with autologous cancellous graft, whereas in the CPG group the defect was filled with CPG solely. After 6 weeks radiological and histomorphometrical analysis showed significantly more new bone formation in the BMC+CPG+PRP group compared to the BMC+CPG group and the CPG group. There were no significant differences between the BMC+CPG+PRP group and the autograft group. In the PRP platelets were enriched significantly about 4.7-fold compared to native blood. In BMC the count of mononuclear cells increased significantly (3.5-fold) compared to the bone marrow aspirate. This study demonstrates that the composite of BMC+CPG+PRP leads to a significantly higher bone regeneration of critical-size defects at the proximal tibia in mini-pigs than the use of BMC+CPG without PRP. Furthermore, within the limits of the present study the composite BMC+CPG+PRP represents a comparable alternative to autologous bone grafting.

## Introduction

The autologous cancellous graft represents up to this date the therapeutical gold standard in the treatment of bone defects on long bones [1]–[3]. This, however, often results in a high donor site morbidity and its availability is also limited [4], [5]. During the past years more and more osteoconductive bone substitutes and osteoinductive growth factors have been used isolated or in combination with supplementary therapeutic options. As a source for these growth factors platelet-rich plasma (PRP) has been described [6], [7]. The effects of which, however, are being discussed controversially in literature [8]–[13]. At an increasing rate cell therapeutics, such as mesenchymal stem cells from bone marrow in combination with osteoconductive bone substitutes are being used today [7], [14], [15]. One possible application method of cell therapeutics is the use of bone marrow concentrate (BMC), which is obtained by density gradient centrifugation [16], [17]. In spite of the various promising results of the sole or combined use of the osteoconductive and osteoinductive substances described before, their effectiveness is still inferior to autologous cancellous grafts [14], [18]. The combination of an osteoconductive bone substitute with PRP and BMC, however, could mean a further step in the search of a composite with an osteogenetic potential equivalent to autologous bone grafts. In this context Marx et al. and Dallari et al. were able to show that the growth factors in PRP, when serving as mitogenes, can only stimulate the growth of preexisting vital bone cells or osteogenic cells and therefore have to rely on their local existence [6], [11]. So the addition of autologous mesenchymal stem cells in the form of BMC could provide an increased presence of osteoprogenitor cells. These would then be available for the stimulation of the mitogenous growth factors in PRP. The PRP effect on bone healing is thought to be mainly dependent on the proliferation promoting function, with the molecular mechanisms largely unknown [19]. Several authors demonstrated that PRP promotes the proliferation of human MSCs as well as rat-derived MSCs and mouse-derived MSCs in vitro [19]–[22].

The object of this animal study was to evaluate whether an additive application of PRP to a composite made of BMC and calcium phosphate granules (CPG) can positively influence new bone formation during the early phase of the bone healing process. Furthermore this study should find out if the total composite of BMC+CPG+PRP could possibly be a substitute for autologous bone grafting.

## Materials and Methods

### Animals and Ethics Statement

32 female Goettinger mini-pigs (aged 20–28 months, weight 24–32 kg) were used in this study. Animals were treated in compliance with our institution’s guiding principles ‘in the care and use of animals’ and in accordance with the EU Directive 2010/63/EU for animal experiments. The local ethics committee for animal experiments (LANUV NRW, Recklinghausen, Germany) approved the design of the experiment (Permit Number: 8.87-50.10.37.09.253). A priori power analysis using bone regeneration data from previous experiments with a similar animal model was performed. This resulted in a sample size of 8 for a power of 80% with a p value of 0.05 determining significance [12].

### Animal Model and Surgery

All animals were starved for a minimum of 12 h before surgery. Antibiotic preparation was given once to each animal in a perioperative way as single shot of 3.3 ml Lincomycin (Lincomycin 20%, WDT, Garbsen, Germany). After intramuscular sedation with 0.5 mg/kg Atropin (Atropinsulfat, B Braun, Melsungen, Germany), 5 mg/kg Azaperon (/Stresnil, Janssen-Cilag GmbH, Neuss, Germany) and 10 mg/kg Ketamin (Ketavet, Pharmacia GmbH, Karlsruhe, Germany), anaesthesia was initiated using 0.5 g Thiopental (Inresa Arzneimittel GmbH, Freiburg, Germany). For all surgical procedures, inhalation anaesthesia was performed by use of oxygen and nitrous oxide and isoflurane. To maintain hydration and cardiac protection, all animals received a constant rate infusion of 5% Glucose’s solution (Delta-Select, Pfullingen, Germany) combined with 10 ml Inzolen (Koehler Chemie GmbH, Alsbach-Hähnlein, Germany) and 5 ml Lidocain 2% (Lidocain-HCl, B. Braun, Melsungen, Germany) while anaesthetized. Intraoperative analgesia was performed by intravenous injection of 0.4 mg/kg Piritramid (Dipidolor, Janssen-Cilag GmbH, Neuss, Germany) and 4.5 mg/kg Carprofene (Rimadyl, Pfitzer Pharma GmbH, Karlsruhe, Germany). For postoperative treatment, Piritramid and Carprofene were applied subcutaneously for 3 days at the same dose. Additionally, prophylactic administration of Lincomycin (3,3 ml Lincomycin 20%, WDT, Garbsen, Germany) was performed postoperatively for 3 days.

**Figure 1:**
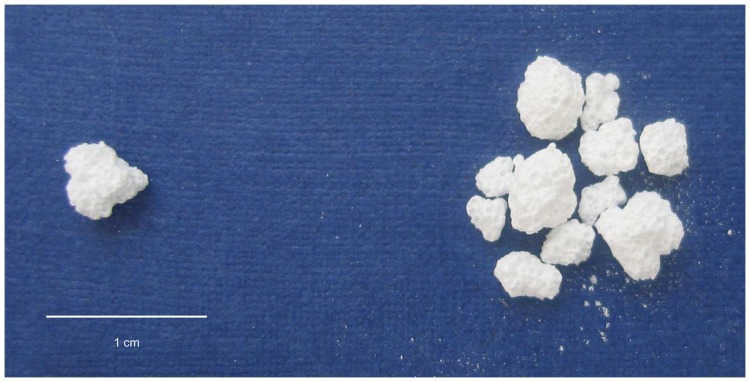
Calcium Phosphate Granules (CPG): spherical, micro- and macroporous (micro: 2–10 and macro: 150–550 µm pores), carbonated, apatic calcium phosphate granules produced from a calcium phosphate self-setting cement (granule size: 2–4 mm, total porosity of 50% or more).

The animals were randomized into the CPG group, BMC+CPG group, the BMC+CPG+PRP group and the autograft group. Each group consisted of eight mini-pigs. According to the animal model developed by Hakimi et al. [12] a cylindrical defect with a diameter of 11 mm from medial to lateral to a depth of 25 mm at the right proximal tibia without lateral cortical penetration was surgically created in all animals using a cannulated reamer (Aesculap AG & Co. KG, Tuttlingen, Germany). In order to insure the correct depth of the defect the reamer had a marking at 25 mm. After reaming the depth was controlled again by using a depth measuring device. In the CPG group, the BMC+CPG group and the BMC+CPG+PRP group spherical, micro- and macroporous (micro: 2–10 and macro: 150–550 µm pores), carbonated, apatic calcium phosphate granules produced from a calcium phosphate self-setting cement powder comprising α-Ca3(PO4)2 (61 wt.%), CaH- PO4 (26%), CaCO3 (10%) and precipitated hydroxyapatite, Ca10(PO4)6(OH)2 (3%) (CPG, granule size: 2–4 mm, total porosity of 50% or more, Calcibon Granules, Biomet Deutschland GmbH, Berlin, Germany) were used as scaffolds **(** **Fig. 1** **)**. In these groups 2.4 cm^3^ of CPG (equates app. 0.95 g) were used to fill the defect.

All surgical procedures took place under general anaesthesia and aseptic conditions and were performed by the same experienced surgeon. Moreover, all procedures were conducted in a single surgery. The right proximal tibia was exposed using a medial approach and soft tissues were reflected. The aforementioned defect was created 10 mm distal to the joint line and 12 mm anterior to the most posterior aspect of the tibia. In the CPG group the defects were filled with CPG solely. Prior to implantation, in the BMC+CPG+PRP group, the calcium phosphate granules (CPG) were soaked with autologous PRP for five minutes. Then the consistency of the BMC+CPG+PRP composite was gel-like allowing adaptation of the biomaterial to the complex geometry of the bone defect. In the BMC+CPG group the calcium phosphate granules (CPG) were soaked with BMC for five minutes and then implanted into the defect. In the autograft group an incision was made over the left iliac crest and sharp dissection was used to expose the bone. A Kirschner guide wire (K-wire) was inserted in the iliac crest. Then, the cancellous graft chips were harvested from the iliac crest using a cannulated reamer of 11-mm diameter inducted over the guide wire. In the autograft group the defect was filled only with autologous cancellous graft chips. The total volume of this graft chips was identical (2.4 cm^3^) to the proximal tibial defect created before. Soft tissues were closed in layers.

### Bone Marrow Harvest

In the BMC+CPG and the BMC+CPG+PRP group bone marrow (BM) was harvested from the iliac crest by Jamshidi vacuum aspiration. From the bone marrow aspirate the mononuclear cells were concentrated into bone marrow concentrate (BMC) using a point-of-care device (MarrowStim mini concentration system, Biomet Biologics, Inc., Warsaw, Indiana, USA).

According to the protocol of the manufacturer 6 ml of citrate anticoagulant (ACD-A, Anticoagulant Citrate Dextrose Solution) were added to 2×12 ml of bone marrow from both iliac crests. This mixture was centrifuged at 600×g for 15 min and 3–4 ml of BMC was separated from the cell free plasma/ACD-A mixture and from the red blood cells. Then an average of 3.5 ml±0.5 of BMC was separated from the cell free plasma/ACD-A mixture and from the red blood cells according to the manufacturer’s instructions. 0.5 ml of the BMC was used for in-vitro analysis. 1.2 ml of BMC was soaked by CPG and then implanted into the defect.

The concentration of mononuclear cells in the BM aspirate and in the separated BMC was analyzed by an automatic cell counter using veterinary software (ADVIAs 120, Bayer Diagnostics GmbH, Leverkusen, Germany). According to Herten et al. [23] the concentration factor of the BMC was calculated from the quotient of mononuclear cells in BM/BMC.

### Postoperative Management and Sacrifices

All animals were fully weight bearing immediately after surgery. After 6 weeks (42 days) postoperatively the mini-pigs were sacrificed by an overdose of sodium pentobarbital 3% (Eutha 77, Essex Pharma GmbH, München, Germany). The proximal tibia was dissected from the distal femoral shaft to the proximal tibial shaft. All specimens were fixed in 10% neutral buffered formalin solution for 14 days.

### Preparation of Platelet-rich Plasma (PRP)

In the BMC+CPG+PRP group PRP was prepared according to the instructions of the manufacturer from 120 ml autologous whole blood. The blood was retrieved from the jugular vein of the mini-pig directly prior to surgery. PRP was prepared with the „GPS II Platelet Separation System“ (Biomet Biologics, Warsaw, IN, USA). To 6 ml of citrate anticoagulant 54 ml of autologous whole blood was added and centrifuged at 600×g for 15 minutes. The platelet-poor plasma (PPP) was separated resulting in 20 ml of PRP. For the activation of the platelets, autologous thrombin was produced from 7 ml of whole blood by centrifugation and used in combination with 2 ml of 10% calcium chloride. The thrombocyte concentrations in whole blood and the resulting PRP were analyzed in an automatic counter as described above. The concentration factor of the PRP was calculated from the quotient of the thrombocyte count in whole blood and PRP. As recommended by the manufacturer the concentrations of PDGF-bb were quantified in serum and PRP, whereas the concentrations of TGFβ1 were quantified in plasma and in PRP using a commercially available ELISA (Quantikine ELISA-Kits, R&D Systems, MN, USA).

### MSC Culture

The mononuclear cells from BM and from BMC were cultured in standard cell culture medium (Dulbecco’s modified Eagle medium, DMEM), with 1 g/l glucose, 20% fetal bovine serum (FBS), 100 units/ml penicillin, 100 µg/ml streptomycin and 2 mM glutamine. All ingredients were obtained from PAA Laboratories GmbH, Pasching, Austria. Culture conditions were 5% CO_2_ and 37°C.

### CFU Assay

The proliferation potency of the cells was measured in colony-forming units (CFU). Equal concentrations of mononuclear cells from BM and from BMC were seeded in 24 - well plates in different cell densities in order to reach a clonal dilution. Cells were seeded at a density of 5×10^5^, 1×10^5^, 5×10^4^ and 1×10^4^ cells/cm^2^ with n = 3 wells per density for CFU-F and CFU-ALP, respectively. After 14 days in culture the cells were fixed in formalin, rinsed with phosphate buffered saline (PBS) and incubated for 60 min at room temperature with either hematoxylin in order to determine the number of CFU-fibroblast-like cells (CFU-F) or substrate for the alkaline phosphatase enzyme (ALP) in order to identify the osteogenic regeneration potential CFU-ALP.

### Cell Characterization

For phenotypic characterization adherent cells of the first passage were used for FACS analysis of typical MSC differentiation markers as described in detail by Herten et al. [23].

For Lineage Differentiation the cells were further cultivated for osteogenic, chondrogenic and adipogenic differentiation as demonstrated previously [23].

### Radiological Evaluation

#### 1. Multidetector-CT (MD-CT)

CT examinations of the explanted tibial bones after 6 weeks (42 days) were performed on a 64-detector row CT scanner (SOMATOM sensation Cardiac 64, Siemens Medical Solutions, Germany). A collimation of 64×0.6 mm with 1.0 s rotation time and a pitch factor of 0.8 were chosen at a tube potential of 120 KV and an effective tube current-time product of 180 mAseff. For image analysis, axial overlapping slices of 0.6 mm were reconstructed (reconstruction increment: 0.4 mm) using a bone (B70f) convolution kernel. Images were documented in a bone window (center 700/width 4000 Hounsfield Units [HU]). Additionally, primary overlapping coronal and sagittal multi-planar reformations (MPR) with 0.6 mm slice thickness were reconstructed. For further analysis, all data were transferred to a multimodality workstation (Siemens Medical Solutions, Germany). The volume of the bone defect and the extent of bone healing were measured quantitatively using a commercially available software program on the multi-modality workstation (Syngo Volume, Siemens Medical Solutions, Germany):

The workstation enables volume measurements of the tissue of interest with respect to density (Hounsfield Units [HU]). According to Riegger et al. axial images were used for the volumetric measurements [24]. Two board-certified radiologists outlined the entire defect zone manually in consensus in several axial slices. The gaps in between those slices were interpolated by the software automatically and double-checked by the radiologists. Based on the mean CT-values of cortical and trabecular bone a threshold value of 500 HU for osseous consolidation was defined. Then, the volume of the defect was measured three times including different HU ranges [24]:

1. Overall size of defect: measured by including all pixels containing HU from −100 to +3000.
2. Areas of consolidation: measurement of pixels with densities between 500 and 3000 HU.
3. Non-consolidated areas: measurement of all pixels with densities between −100 and 500 HU.

#### 2. Quantitative Cone Beam CT (CBCT) –volumetry

CBCT examinations were performed 6 weeks (42 days) after the bone defect was created prior to the histological analysis on a CBCT scanner with flat panel detector (PaX-Duo3D, Vatech, Korea). A tube potential of 85 kVp and a tube current of 4 mA were used at a field-of-view (FOV) of 5×5 cm. Scan time was 15 seconds, in-plane voxel size was 0.2×0.2 mm. For image analysis, 624 axial images of 0.08 mm slice thickness were reconstructed using standard software (Ez3D2009, Vatech, Korea). Images were digitally transmitted and stored in the institutional Picture Archiving and Communication System (PACS).

The volume of the bone defect and the extent of bone defect healing were measured quantitatively on a commercially available DICOM-viewer (Osirix Imaging software, 64Bit extended version, Pixmeo, Geneva, Switzerland). This software enables volumetric measurements with respect to the density values of the tissue of interest. All pixels with density values within pre-defined thresholds were selected semi-automatically and marked in colour. Axial images were used for the volumetric measurements. Two board-certified radiologists outlined the entire defect zone manually in consensus. As density values of CBCT differ substantially from CT-values [25], a threshold value of 2350 was defined for osseous consolidation based on mean density values of cortical and trabecular bone [26].

Then, the volume of the defect was measured three times including different settings [26]:

1. Overall size of defect: measured by including all pixels of the outlined defect.
2. Areas of consolidation: measurement of pixels with densities >2350.
3. Non-mineralized areas: measurement of all pixels with densities <2350.

Absolute volumes of consolidation and relative extent of bone regeneration were recorded.

### Histological Preparation of the Bone Segments

The specimens were dehydrated using ascending grades of alcohol and xylene, infiltrated and embedded in methylmethacrylate for non-decalcified sectioning. From each specimen serial sections were cut in the axial direction using a diamond wire saw (Exakt, Apparatebau, Norderstedt, Germany). The sections were ground to a final thickness of approximately 50 µm and stained with toluidine blue.

### Histomorphometrical Analysis

**Figure 2:**
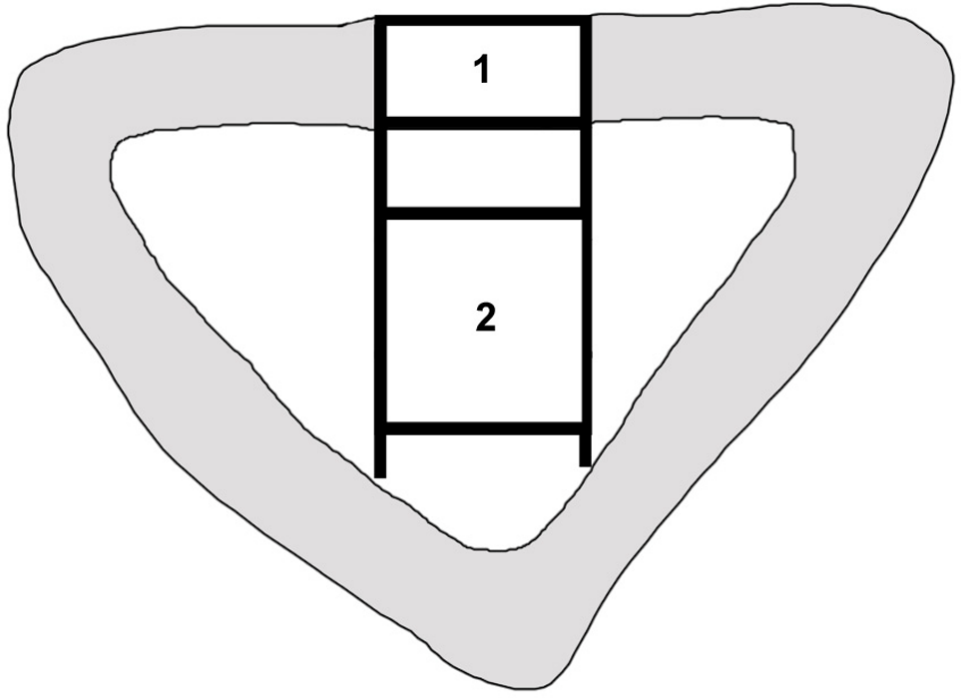
The scheme of a histological slide shows a local bone defect in the proximal tibia. New bone formation was quantified histomorphometrically in two distinct regions of interest. 1. The cortical defect zone (size: 1500×7000 pixels). 2. The central defect zone (size: 3000×7000 pixels).

Histomorphometrical analyses as well as microscopic observations were performed by two experienced investigators in consensus blinded to the specific experimental conditions. For image acquisition a colour CCD camera (Colour View III, Olympus, Hamburg, Germany) was mounted on a binocular light microscope (Olympus SZ 61, Olympus). Digital images (original magnification×6.7) were evaluated using a software program (Cell D, Olympus GmbH, Hamburg, Germany) [27], [28]. The quantitative analysis of new bone formation (area of new-bone formation in µm^2^ and percentage of total new-bone formation) was measured in two predefined regions of interest. One region was positioned in the cortical defect zone (size: 1500×7000 pixels) and the other one in the central area (size: 3000×7000 pixels) of the defect [12] **(** **Fig. 2** **)**. The type of tissue was identified manually, marked and assigned to a colour. In detail, the areas of newly formed bone, connective tissue, and CPG were calculated per total bone defect area. Three sections were evaluated for each bone defect.

### Statistical Analysis

The statistical analysis was performed using SPSS (21.0, SPSS Inc., Chicago, IL, USA). Mean values and standard deviations were calculated. The outcome measures of the radiological and histomorphometrical evaluations were examined by one-way analysis of variance (ANOVA). Differences between the independent variables were checked in post hoc tests [Tukey’s HSD (Honestly Significant Difference) tests for variables]. Significance was defined as a p value <0.05.

For evaluating the outcome measures of the PRP preparations (thrombocyte concentrations in native blood vs. in PRP; concentrations of PDGF-bb in serum vs. in PRP; concentrations of TGFβ1 in plasma vs. in PRP) (comparison of 2 groups) and BMC preparations (concentrations of mononuclear cells in BM aspirate vs. in separated BMC) (comparison of 2 groups) the unpaired student’s t-test was used, p<0.05 was considered statistically significant. The same test was also used for the CFU calculations.

Pearson’s rank correlation (coefficient: **r_P_**) was performed in order to correlate the results of CT volumetry (CBCT and MDCT) with the histomorphometrical results (cortical and central defect zone). Significance was defined as a p value <0.05.

## Results

### Platelet Concentration and Growth Factor Concentration in PRP

**Figure 3:**
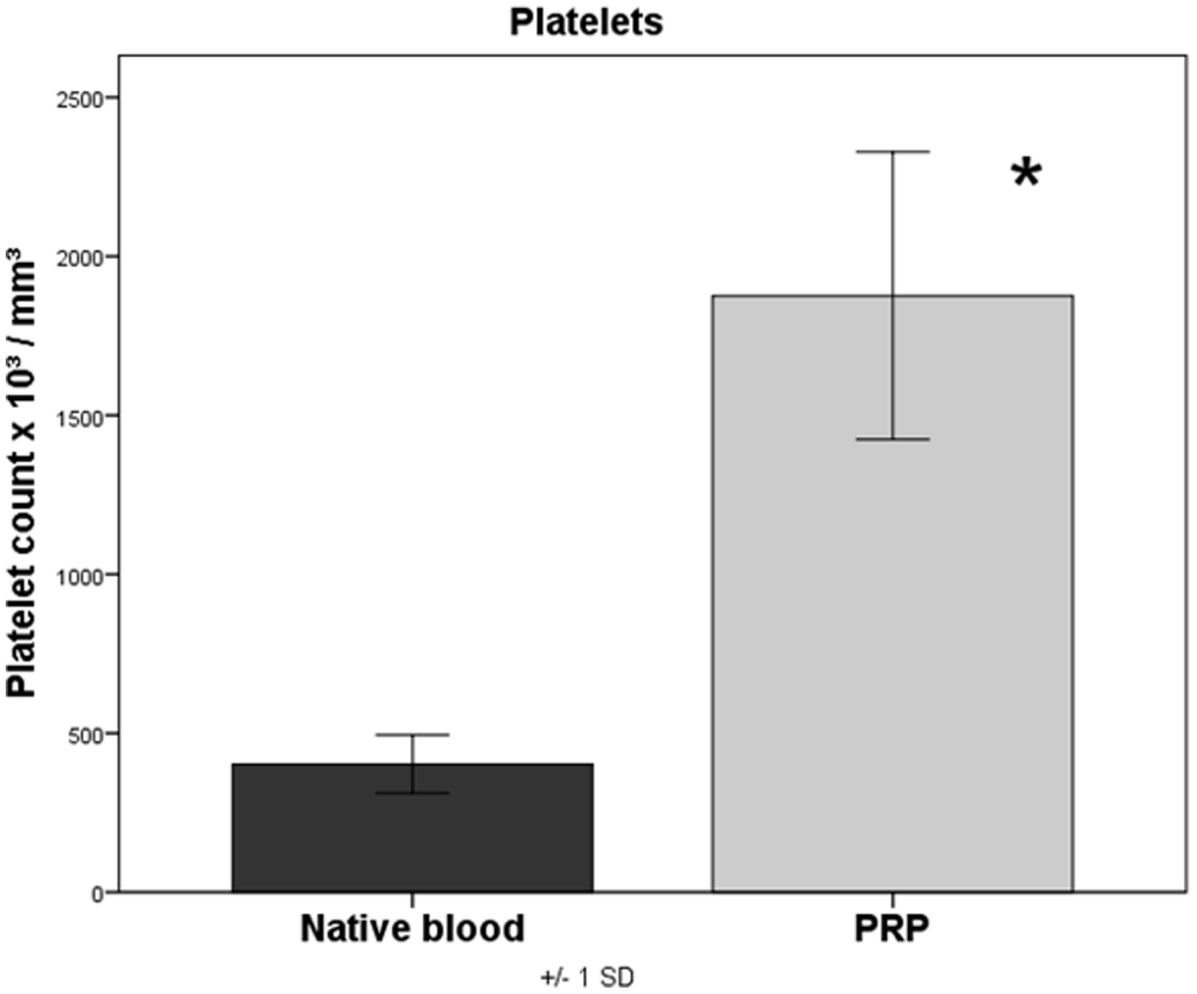
Quantification of platelets in native blood and PRP, p<0.001 PRP versus native blood.

**Figure 4:**
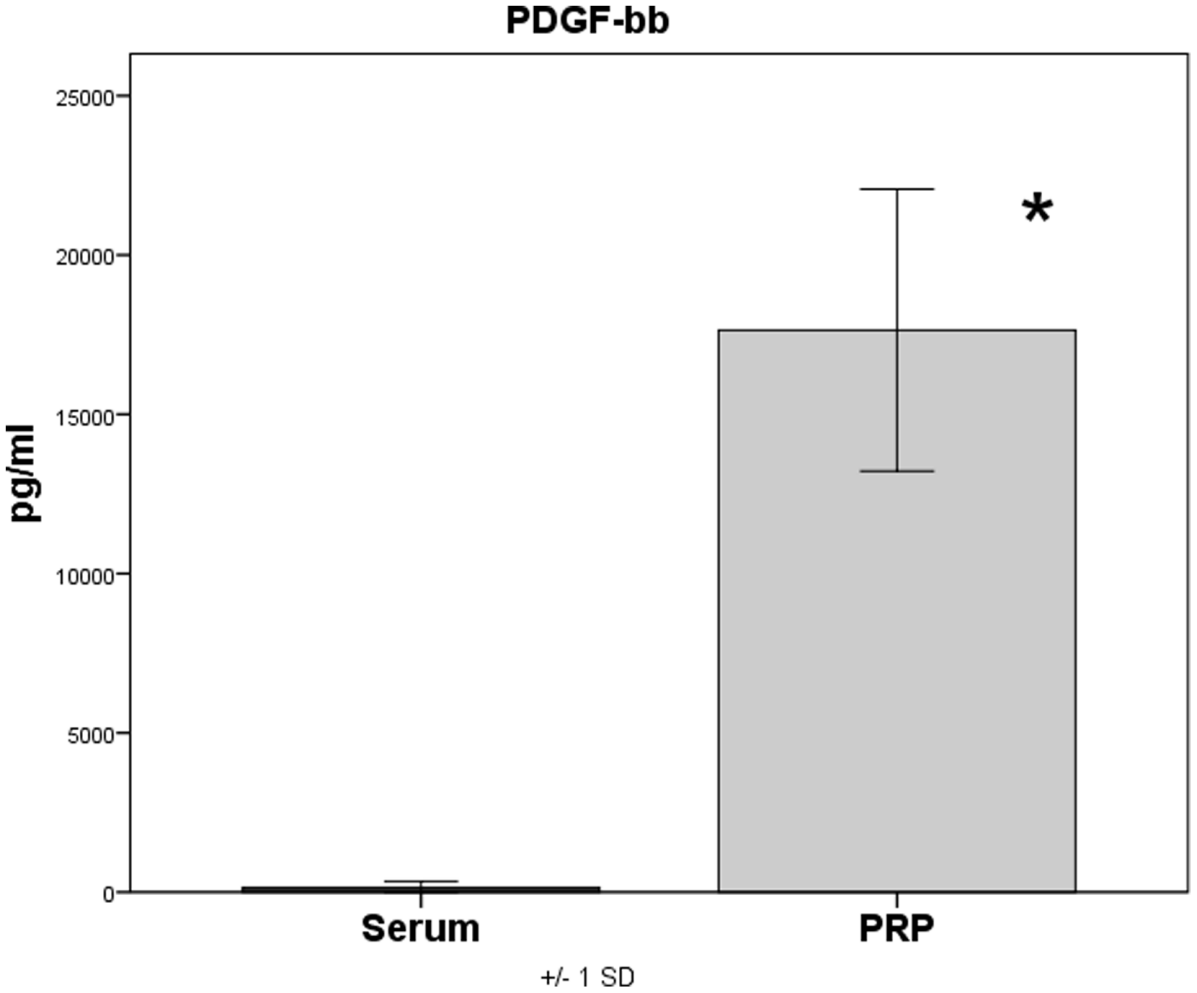
Quantification of PDGF-bb in serum and PRP, p<0.001 PRP versus serum.

**Figure 5:**
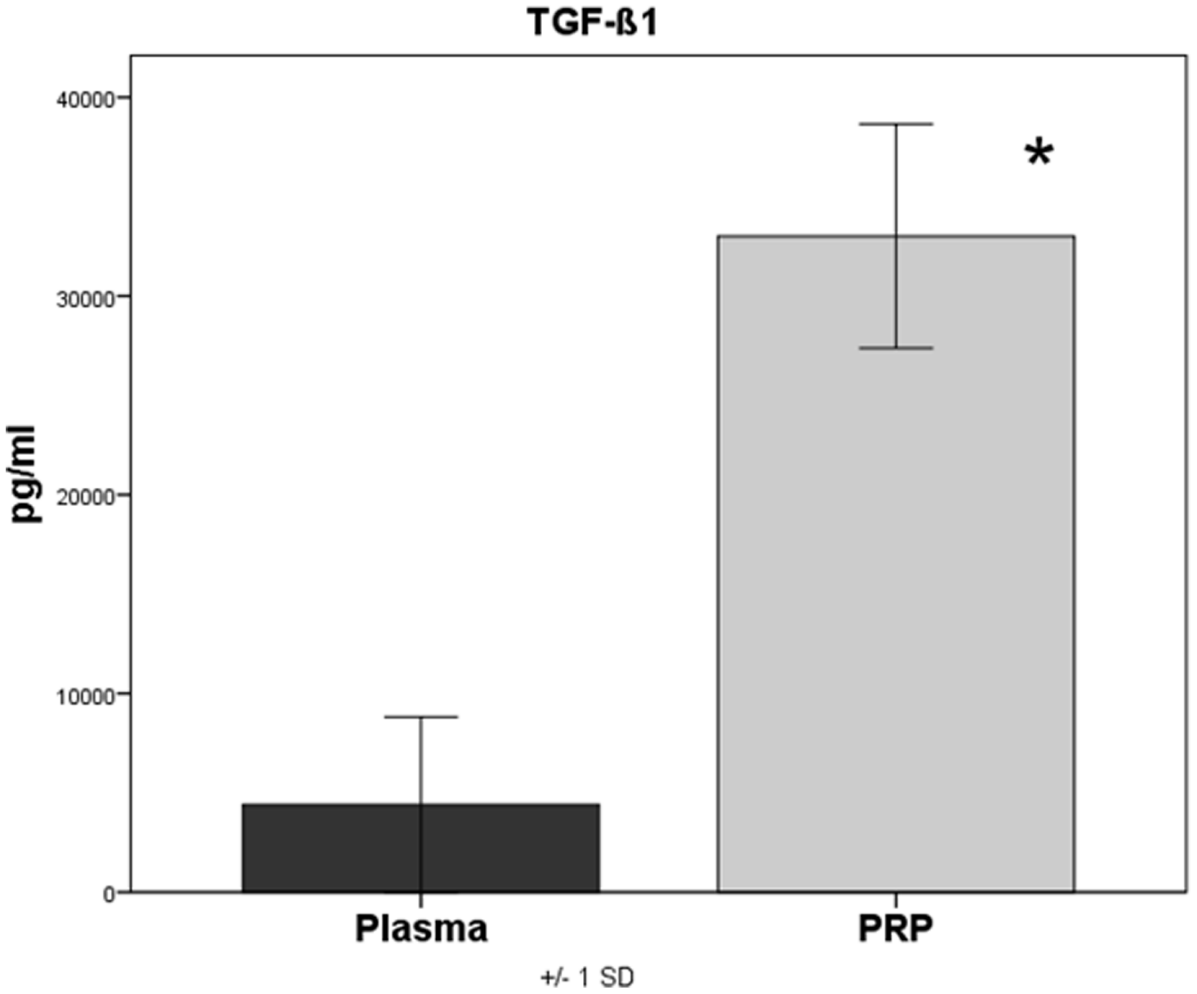
Quantification of TGF-β1 in plasma and PRP, p<0.001 PRP versus plasma.

While the platelet concentration in the native blood interindividually varied between 245 and 520×10^3^/mm^3^ platelets (mean = 398.8×10^3^/mm^3^±91.2), the platelet concentration in PRP measured between 1289 and 2652×10^3^/mm^3^ (mean = 1879.5×10^3^/mm^3^±452.1). This corresponds to a significant concentration increase (p<0.001) of the platelets by the factor 4.7 in PRP as compared to native blood **(** **Fig. 3** **)**. The PDGF-bb concentrations in serum of native blood measured between 15 and 574 pg/ml (mean = 172.9 pg/ml±201.1), in PRP, however, between 12543 and 24789 pg/ml (mean = 17848.4 pg/ml±4427.1). This corresponds to a significant (p<0.001) increase of the PDGF-bb concentration in PRP by 103.2 times **(** **Fig. 4** **)**. The concentration of TGF-β1 in plasma of native blood measured between 1135.7 pg/ml and 12523.6 pg/ml (mean = 4894.1 pg/ml±4407.3) and in PRP between 24543.1 pg/ml and 42029.9 pg/ml (mean = 33063.3 pg/ml±5632.3). Here too a significant increase (P<0.001) of the TGF-β1 concentration in PRP by 6.8 times could be observed **(** **Fig. 5** **)**.

### Concentration Factor of BMC

**Figure 6:**
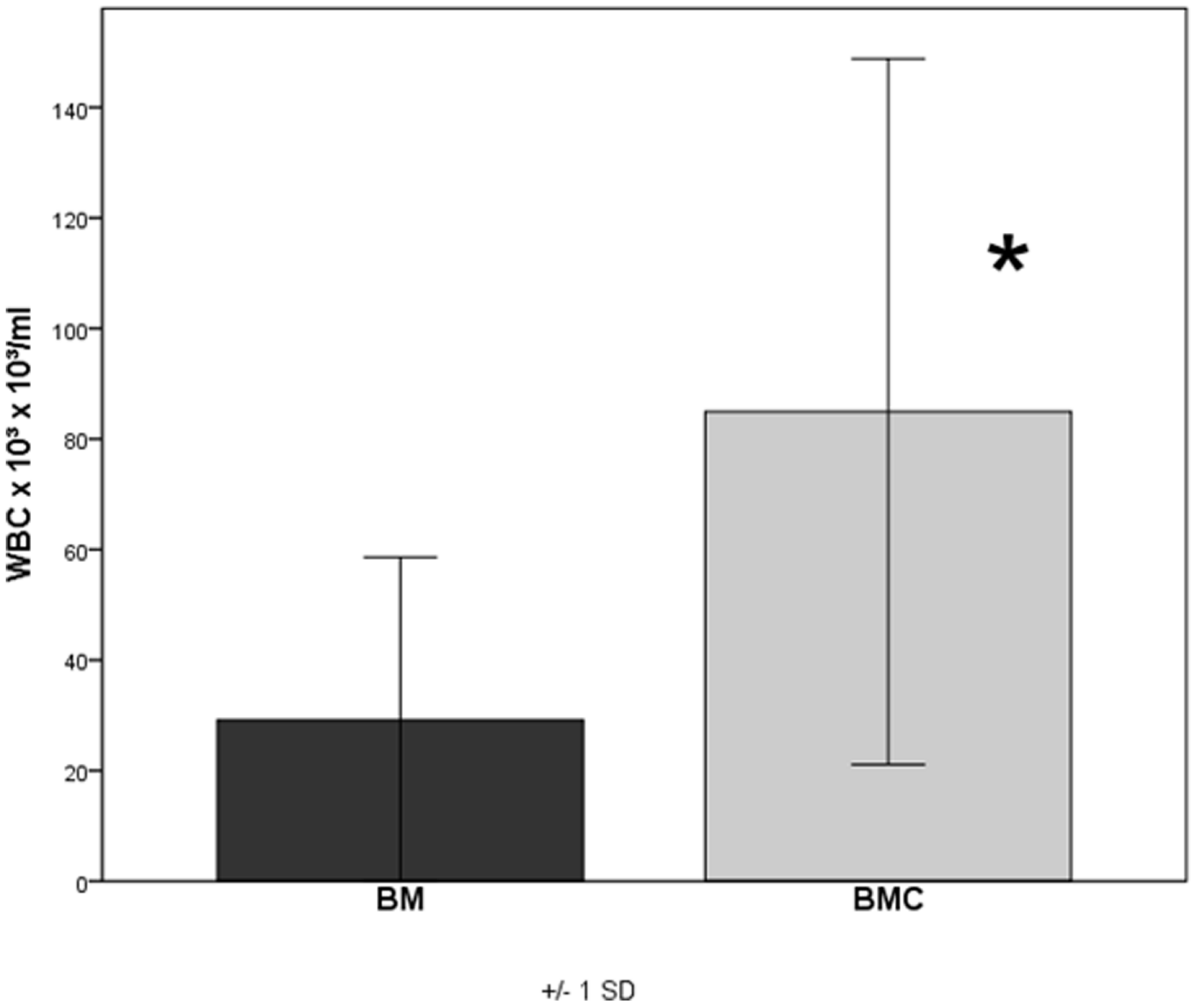
Concentration factor of BMC: The resulting concentration factor of BMC was 3.5±1.39 stating a significant increase of mononuclear cells within the BMC (p = 0.006) compared to the BM.

An average of 29.1×10^6^ cells/ml±28.4 mononuclear cells could be obtained in BM. In the BMC the mean concentration was 89.9×10^6^ cells/ml±61.5 with n = 16. The resulting concentration factor of BMC was 3.5±1.39 stating a significant increase of mononuclear cells within the BMC (p = 0.006) compared to the BM **(** **Fig. 6** **)**. According to these data 1.2 ml×89.9×10^6^ mononuclear cells/ml = 10.8×10^7^ mononuclear cells were implanted into each defect of the BMC+CPG group and the BMC+CPG+PRP group on average.

### Cell Proliferation and Formation of Colonies

**Figure 7:**
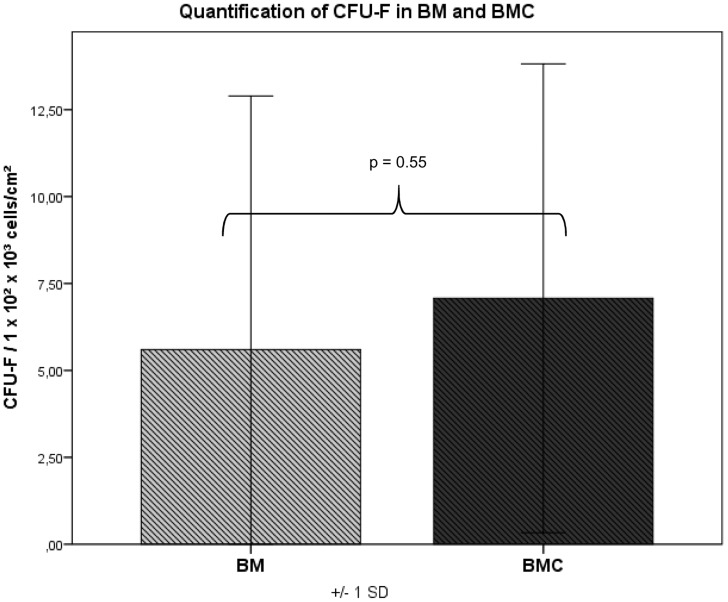
CFU values: in BMC the average CFU-F value was 1.27-fold higher than in BM but without any significance (p = 0.55).

**Figure 8:**
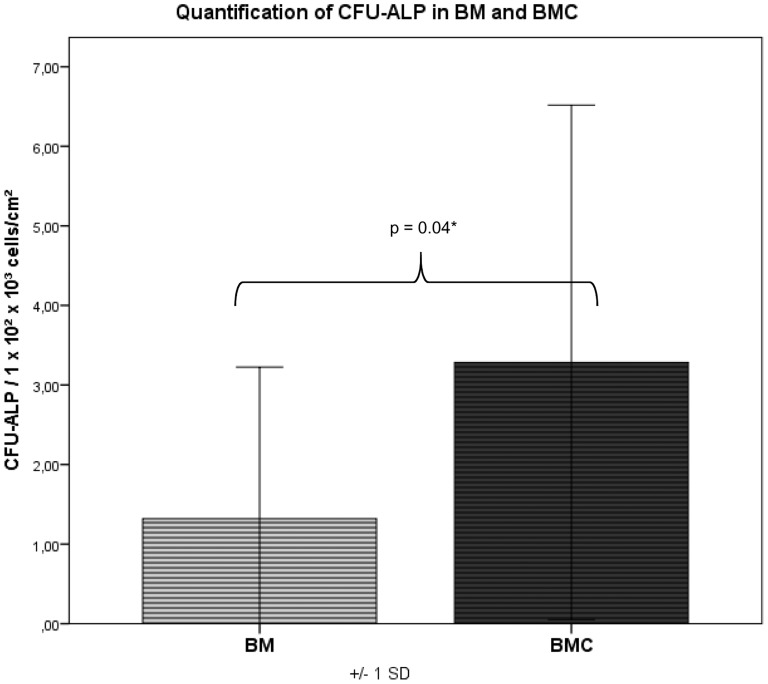
CFU values: the amount of CFU-ALP was 2.5-fold higher and significantly increased in BMC compared to BM (p = 0.04).

**Figure 9:**
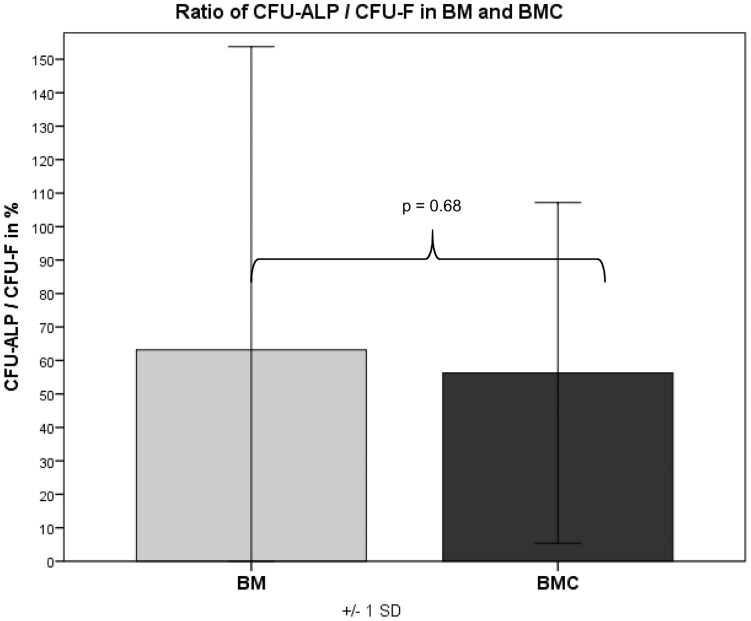
CFU values: the ratio of CFU-ALP/CFU-F was 68% in BM and 56.5% in BMC (without any significant difference, p = 0.68) indicating a comparable differentiation grade into osteoblastic cells within the mononuclear cells of the BM and BMC.

Adherent cells reached confluency after 16–21 days. The amount of CFU-F and CFU-ALP was counted in colonies consisting of more than 40 cells with a defined center. CFU-F/CFU-ALP could be detected in the bone marrow (BM) in 14 out of 16 animals and in the BMC in 15 out of 16 species. After 14 days in BMC the average CFU-F value was 1.27-fold higher than in BM but without any significance (p = 0.55) **(** **Fig. 7** **)**. The amount of CFU-ALP was 2.5-fold higher and therefore significantly increased in BMC compared to BM (p = 0.04) **(** **Fig. 8** **)**. The ratio of CFU-ALP/CFU-F was 68% in BM and 56.5% in BMC (without any significant difference, p = 0.68) **(** **Fig. 9** **)** indicating a comparable differentiation grade into osteoblastic cells within the mononuclear cells of the BM and BMC.

### Cell Characterization

FACS analysis revealed a predominant expression of surface molecules of MSC and a rarer expression of the hematopoetic stem cell surface molecule as demonstrated previously [23].

As published before by Herten et al. cells could be differentiated into the osteogenic, chondrogenic and adipogenic lines in all animals [23].

### Radiological Evaluation

#### 1. MD-CT volumetry

**Figure 10:**
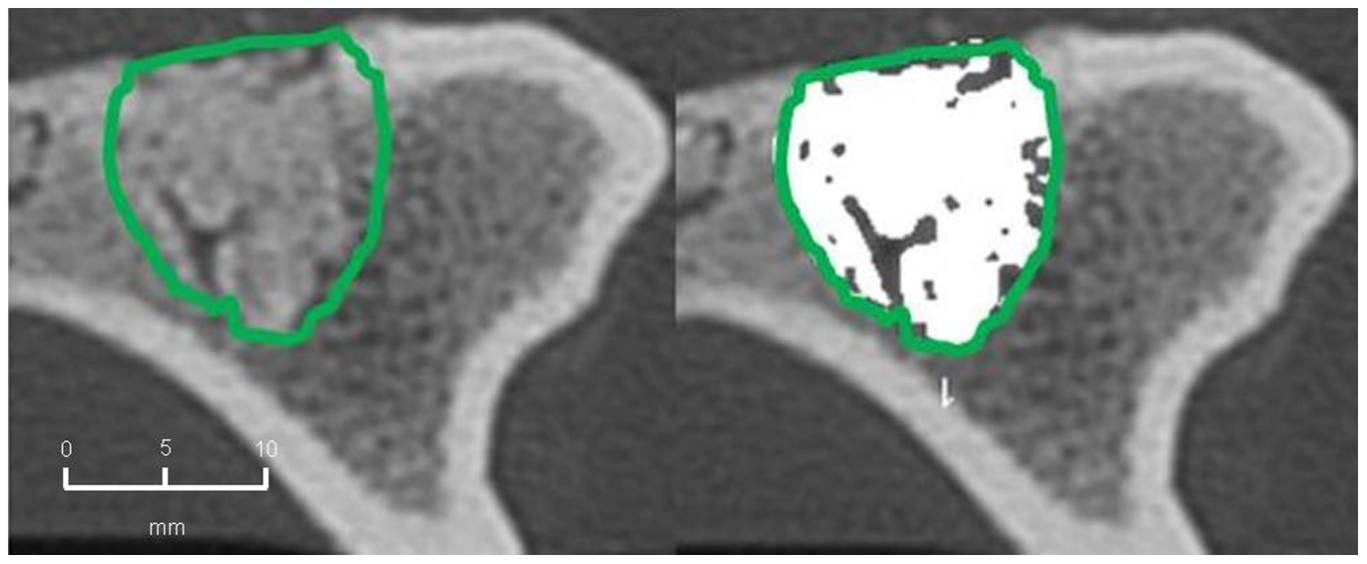
MD-CT volumetry of the tibial defect in axial images: Autograft group: left: defect zone is identified (line), right: only areas with a density >500 HU are opacified (white area), example of an animal with 84% bone defect consolidation.

The mean extent of bone defect consolidation in the BMC+CPG+PRP group was 77.5%±6.7. These results were significantly higher compared to the BMC+CPG (53.5%±19.1, p = 0.001). At the same time the BMC+CPG group was significantly superior compared to the CPG group (26.1%±5.1, p<0.001). The extent of bone healing in the autograft group (81.1%±5.1) did not differ significantly from the BMC+CPG+PRP group (p = 0.906) **(** **Fig. 10** **–** **13** **)**.

**Figure 11:**
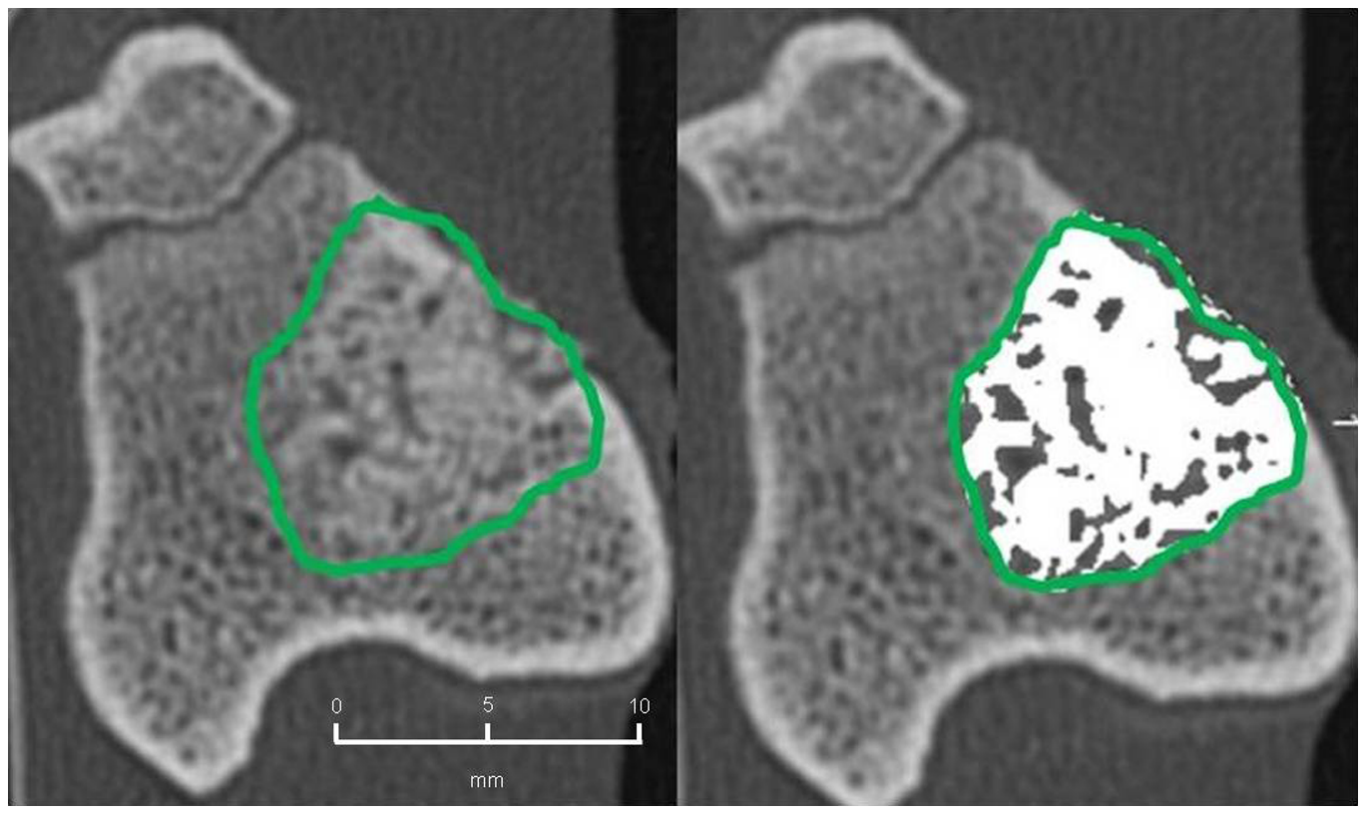
MD-CT volumetry of the tibial defect in axial images: BMC+CPG+PRP group: left: defect zone is identified (line), right: only areas with a density >500 HU are opacified (white area), example of an animal with 79% bone defect consolidation.

**Figure 12:**
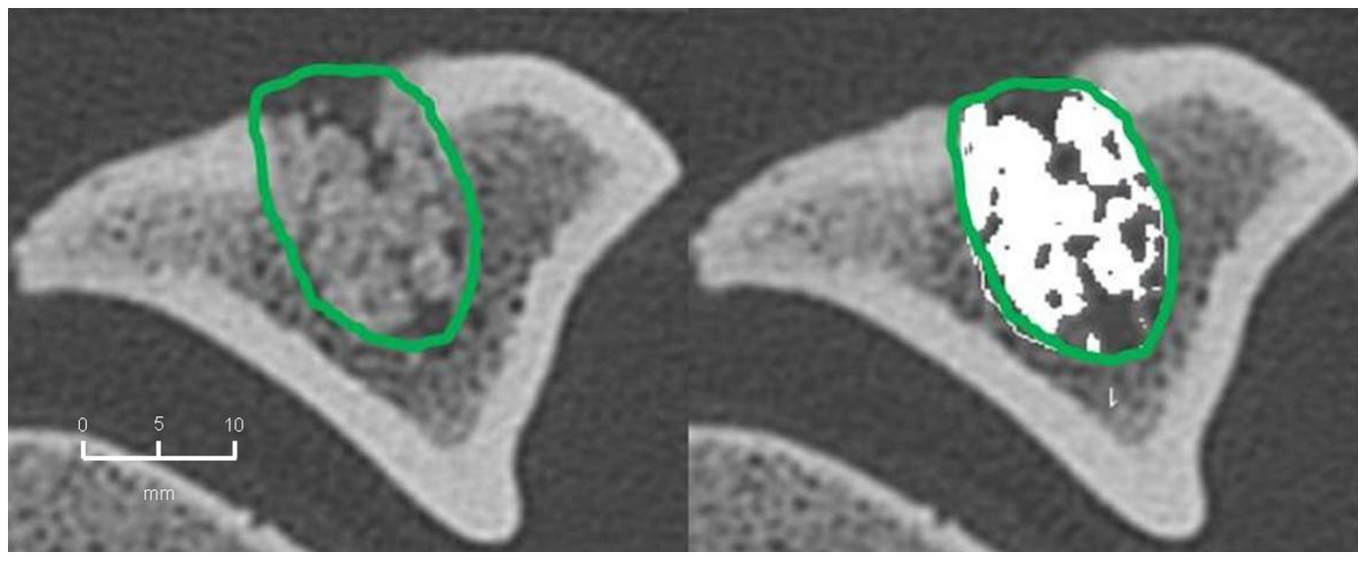
MD-CT volumetry of the tibial defect in axial images: BMC+CPG group: left: defect zone is identified (line), right: only areas with a density >500 HU are opacified (white area), example of an animal with 53% bone defect consolidation.

**Figure 13:**
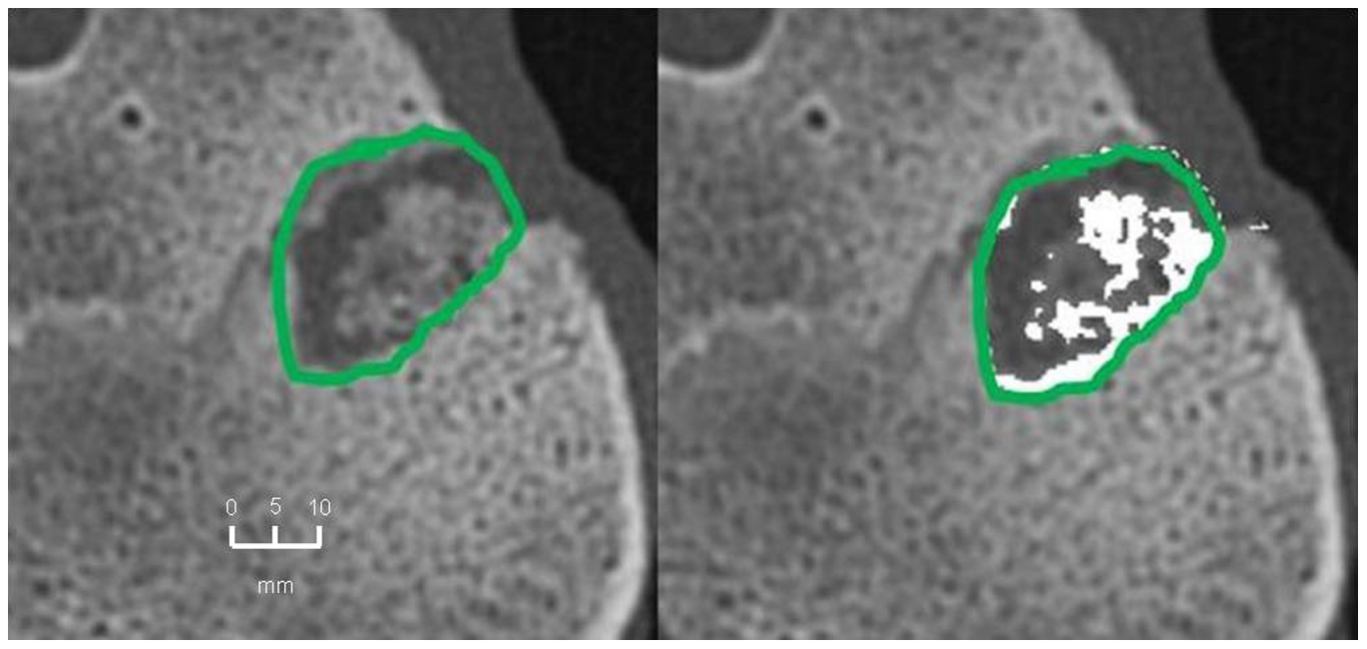
MD-CT volumetry of the tibial defect in axial images: CPG group: left: defect zone is identified (line), right: only areas with a density >500 HU are opacified (white area), example of an animal with 23% bone defect consolidation.

#### 2. CBCT-volumetry

**Figure 14:**
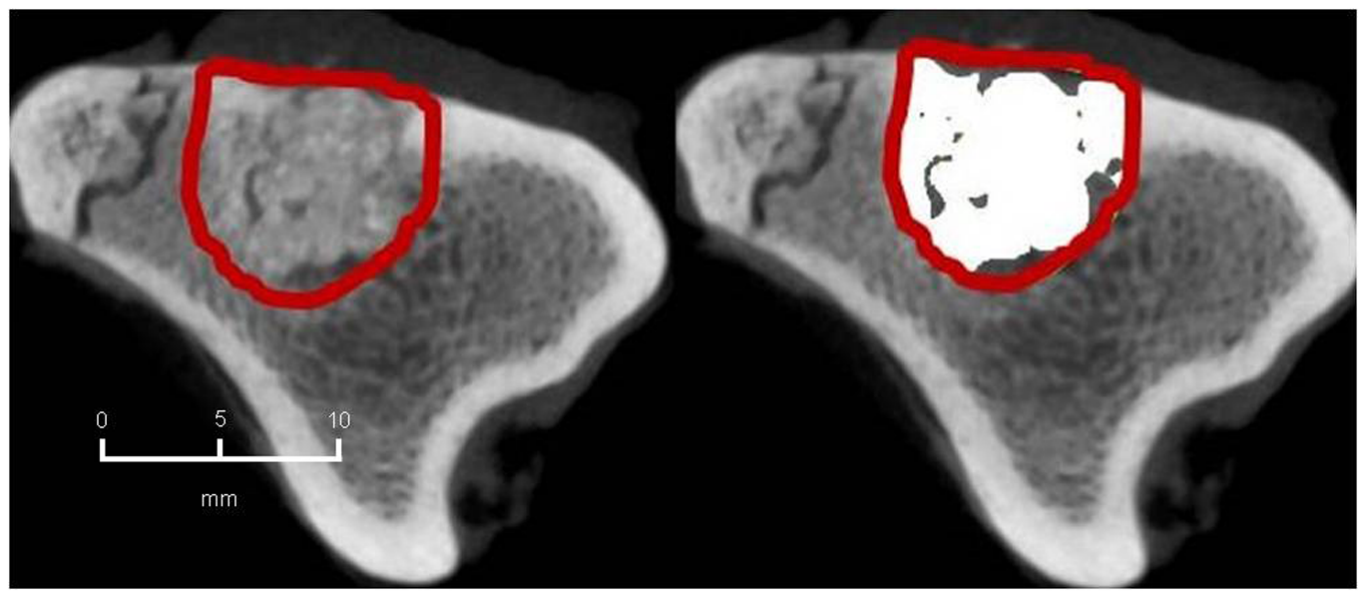
CBCT-volumetry of the tibial defect in axial images: Autograft group: left: defect zone is identified (line), right: only areas with a density >2350 HU are opacified (white area), example of an animal with 83% bone defect consolidation.

In the BMC+CPG+PRP group a mean osseous consolidation of 74.1±9.8% was measured by quantitative volumetry, significantly higher compared to the BMC+CPG (54.7%±12.8, p = 0.001). The BMC+CPG group was significantly superior compared to the CPG group (25.8%±5.3, p<0.001). The autograft group showed an osseous consolidation of 79.5%±5.0. There was no significant difference compared to the BMC+CPG+PRP group (p = 0.613) **(** **Fig. 14** **–** **17** **)**.

**Figure 15:**
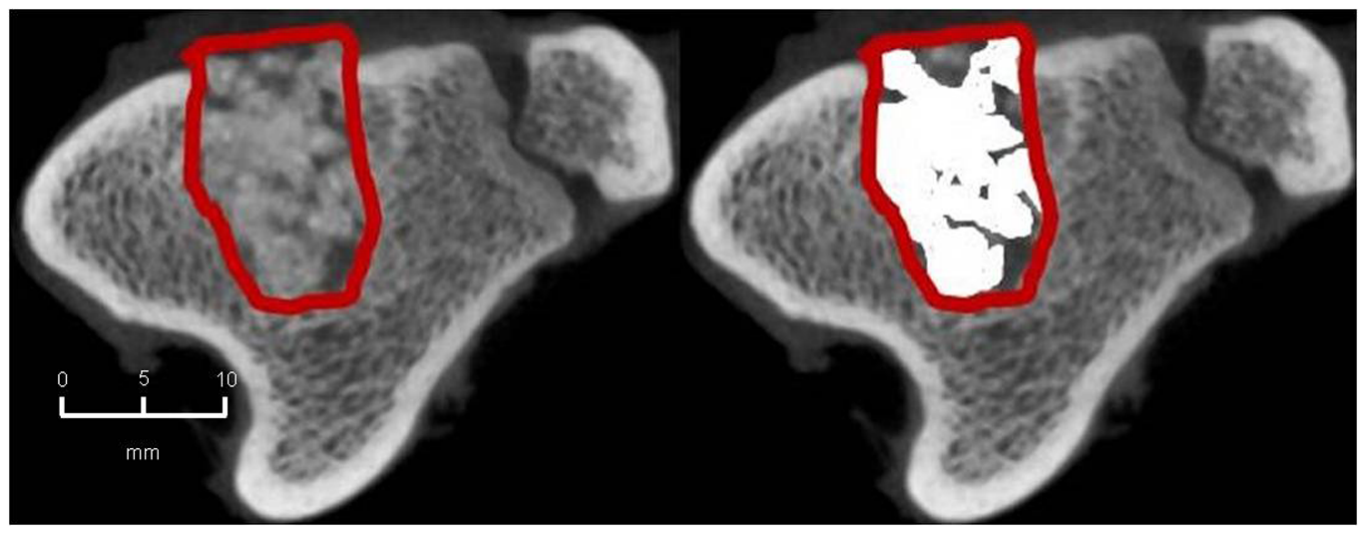
CBCT-volumetry of the tibial defect in axial images: BMC+CPG+PRP group: left: defect zone is identified (line), right: only areas with a density >2350 HU are opacified (white area), example of an animal with 80% bone defect consolidation.

**Figure 16:**
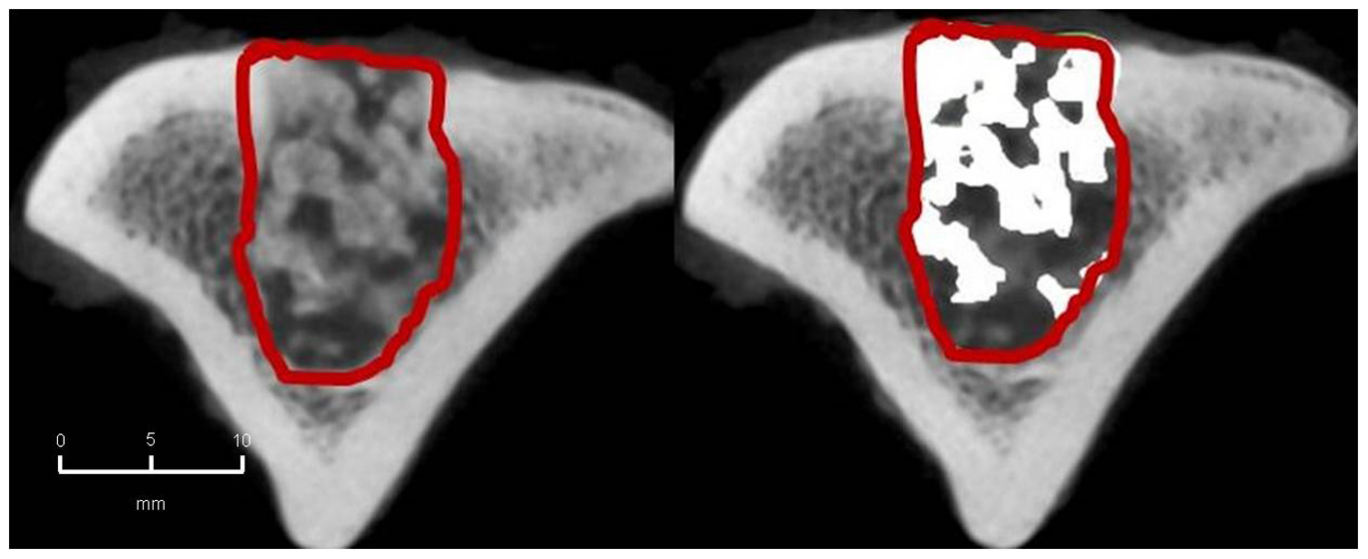
CBCT-volumetry of the tibial defect in axial images: BMC+CPG group: left: defect zone is identified (line), right: only areas with a density >2350 HU are opacified (white area), example of an animal with 54% bone defect consolidation.

**Figure 17:**
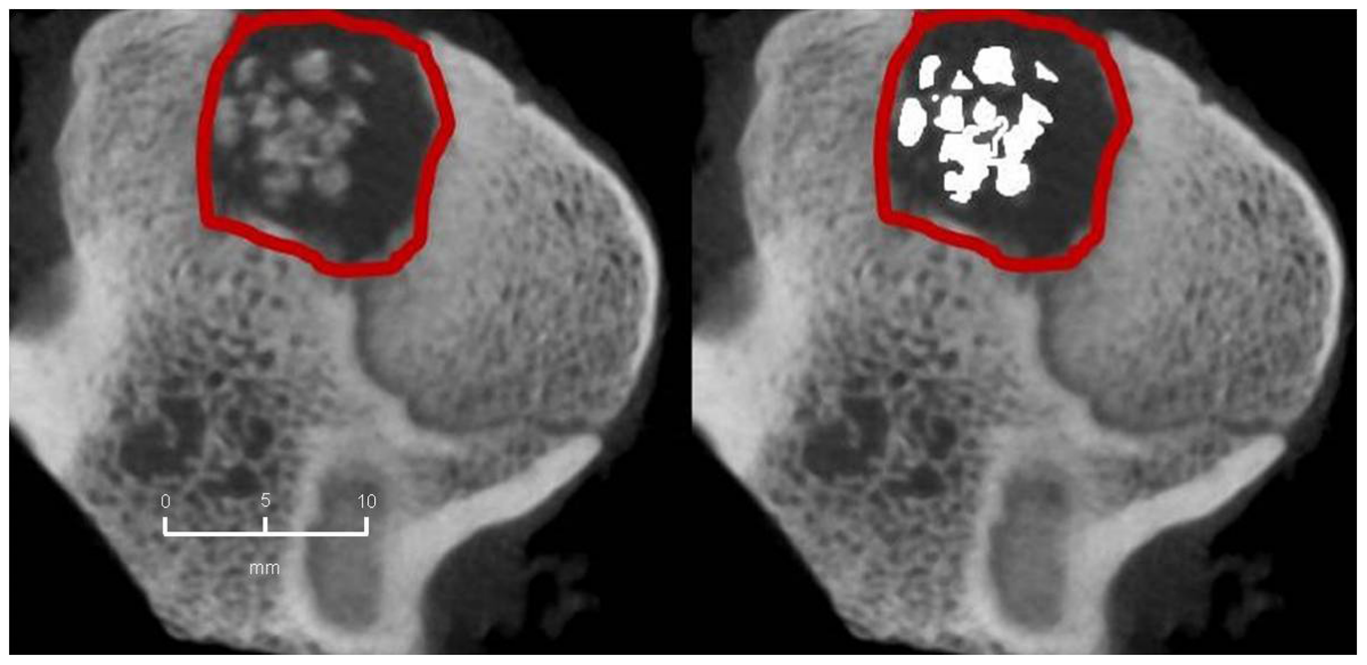
CBCT-volumetry of the tibial defect in axial images: CPG group: left: defect zone is identified (line), right: only areas with a density >2350 HU are opacified (white area), example of an animal with 24% bone defect consolidation.

### Histological Results

**Figure 18:**
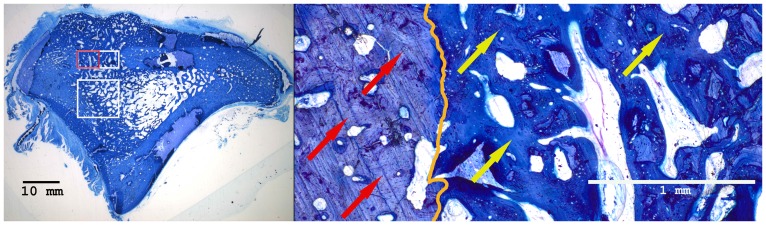
Histological sections of all treatment groups 6 weeks after surgery (left: overview indicating the cortical and central defect zone, rare case of overgrowing new bone formation above the cortical defect zone outside the defect; right: detailed descriptions): Autograft group (detailed description of the cortical defect zone (red rectangle): yellow arrows: newly formed bone (royalblue); red arrows: former cortical bone (purple); orange: borderline between newly formed bone and the former cortical bone).

Histomorphometrical analysis revealed that the area of new bone was significantly larger in the autograft group and the BMC+CPG+PRP group compared to the BMC+CPG group concerning the central area of the defect zone. In the cortical area of the defect zone the autograft and BMC+CPG+PRP group showed a higher new bone formation compared to the BMC+CPG group but the differences were not significant. The autograft group as well as the BMC+CPG+PRP group demonstrated in both areas a significantly higher bone regeneration compared to the CPG group. The BMC+CPG group was centrally statistically superior compared to the CPG group. In the cortical defect zone the BMC+CPG group showed a better but not significantly higher new bone formation. Centrally and cortically there was no difference in the bone regeneration between the autograft group and the BMC+CPG+PRP group (central: p = 0.999; cortical: p = 0.998) **(** **Fig. 18** **–** **21** **and** **22** **/23)**. In only very few histological slides of every treatment group an overgrowth of new bone above the cortical defect zone was observed **(** **Fig. 18** **)**. This overgrowing new bone formation was not quantified due to the rarity of this phenomenon and as it was located outside of the defect. No histological signs of relevant inflammation were found in relation to any of the grafting materials.

**Figure 19:**
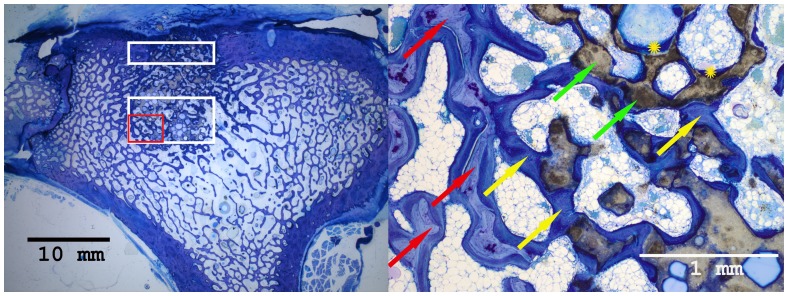
Histological sections of all treatment groups 6 weeks after surgery (left: overview indicating the cortical and central defect zone, rare case of overgrowing new bone formation above the cortical defect zone outside the defect; right: detailed descriptions): BMC+CPG+PRP group (detailed description of the central defect zone (red rectangle): yellow arrows: newly formed bone (royalblue); red arrows: former cancellous bone (light blue); green arrows: non-resorbed remnants of CPG; yellow cross: newly formed bone within already resorbed CPG particles).

**Figure 20:**
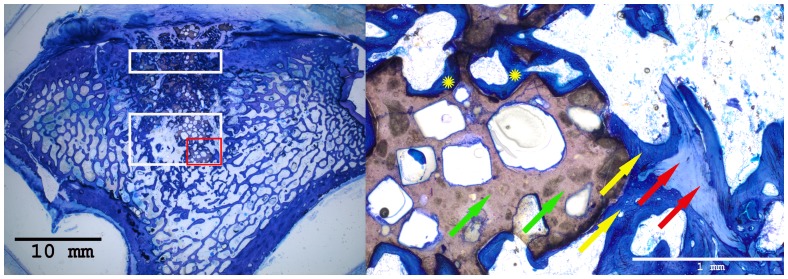
Histological sections of all treatment groups 6 weeks after surgery (left: overview indicating the cortical and central defect zone, rare case of overgrowing new bone formation above the cortical defect zone outside the defect; right: detailed descriptions): BMC+CPG group (detailed description of the central defect zone (red rectangle): yellow arrows: newly formed bone (royalblue); red arrows: former cancellous bone (light blue); green arrows: non-resorbed remnants of CPG; yellow cross: newly formed bone within already resorbed CPG particles).

**Figure 21:**
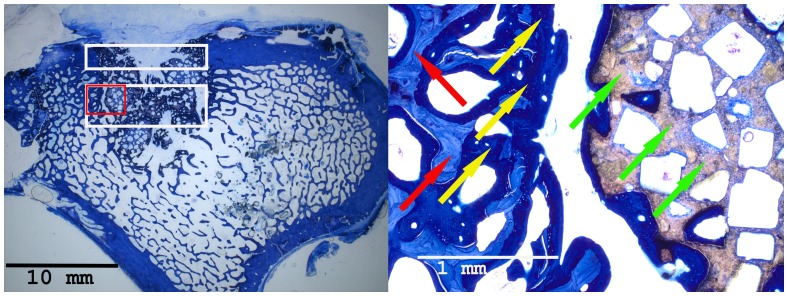
Histological sections of all treatment groups 6 weeks after surgery (left: overview indicating the cortical and central defect zone, rare case of overgrowing new bone formation above the cortical defect zone outside the defect; right: detailed descriptions): CPG group (detailed description of the central defect zone (red rectangle): yellow arrows: newly formed bone (royalblue); red arrows: former cancellous bone (light blue); green arrows: non-resorbed remnants of CPG; yellow cross: newly formed bone within already resorbed CPG particles).

**Figure 22:**
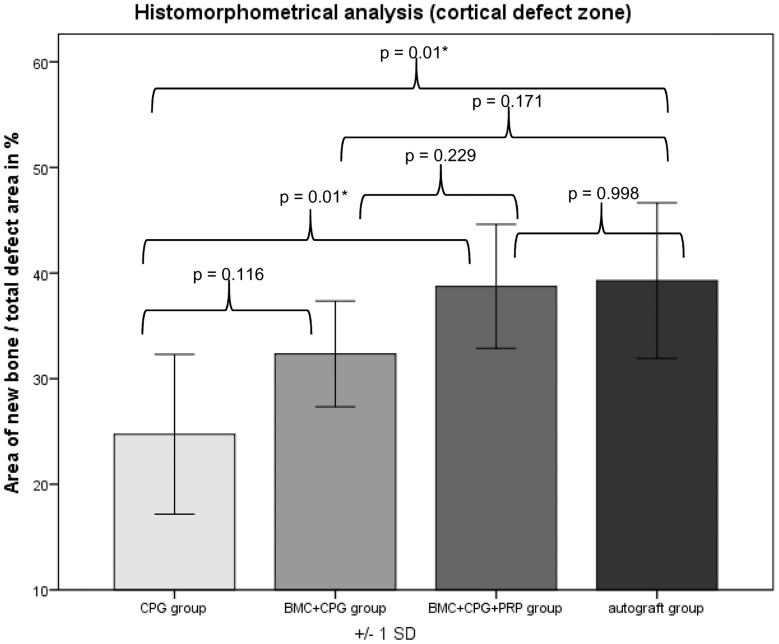
Histomorphometry: Histomorphometrical results of the cortical defect zones.

**Figure 23:**
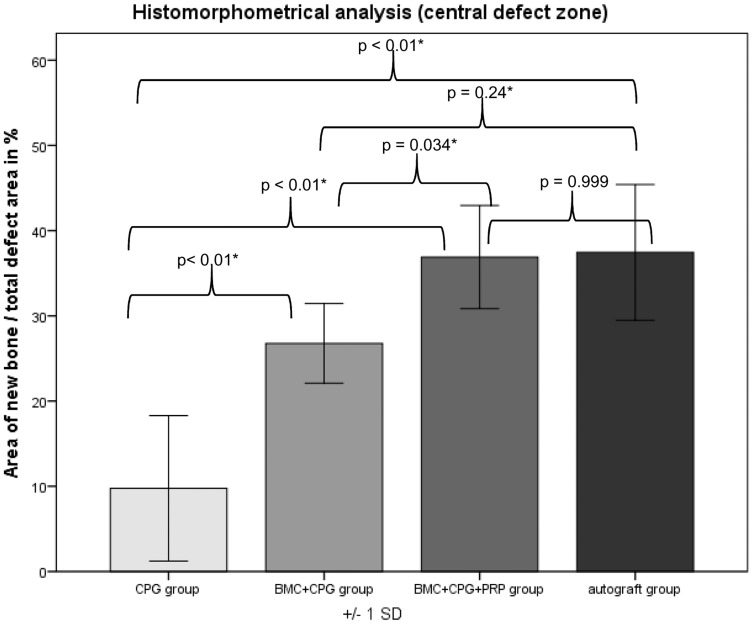
Histomorphometry: Histomorphometrical results of the central defect zones.

### Correlation of the Radiological and Histomorphometrical Result

The histomorphometrical measurements of new bone formation in the cortical as well as the central defect zone of the CPG, BMC+CPG, BMC+CPG+PRP and the autograft group were correlated with the radiologically determined osseous consolidation by MDCT and CBCT. Furthermore, the results of MDCT of every treatment group were correlated with the findings in CBCT.

In all treatment groups both the area of newly formed bone in the cortical and the central defect zone correlated significantly with the findings of MDCT as well as CBCT. Moreover, all MDCT results correlated significantly with CBCT results in all the treatment groups **(** **Tables 1** **–** **4** **)**.

**Table 1:** Correlations Autograft group.

		CBCT	MDCT	Histomorphometrycortical	Histomorphometrycentral
CBCT	Pearson’s rank correlation (coefficient: r)	1	0.995**	0.932**	0.952**
	p - value		<0.001	0.001	<0.0001
	N	8	8	8	8
MDCT	Pearson’s rank correlation (coefficient: r)	0.995**	1	0.915**	0.927**
	p - value	<0.001		0.001	0.001
	N	8	8	8	8
Histmorphometry cortical	Pearson’s rank correlation (coefficient: r)	0.932**	0.915**	1	0.899**
	p - value	0.001	0.001		0.002
	N	8	8	8	8
Histomorphometry central	Pearson’s rank correlation (coefficient: r)	0.952**	0.927**	0.899**	1
	p - value	<0.001	0.001	0.002	
	N	8	8	8	8

**Correlation is significant for p<0,01 (twotailed).

**Table 2:** Correlations CPG group.

		CBCT	MDCT	Histomorphometrycortical	Histomorphometrycentral
CBCT	Pearson’s rank correlation (coefficient: r)	1	0.980**	0.909**	0.833*
	p - value		<0.001	0.002	0.010
	N	8	8	8	8
MDCT	Pearson’s rank correlation (coefficient: r)	0.980**	1	0.947**	0.790*
	p - value	<0.001		<0.001	0.020
	N	8	8	8	8
Histmorphometry cortical	Pearson’s rank correlation (coefficient: r)	0.909**	0.947**	1	0.593
	p - value	0.002	<0.001		0.121
	N	8	8	8	8
Histomorphometry central	Pearson’s rank correlation (coefficient: r)	0.833*	0.790*	0.593	1
	p - value	0.010	0.020	0.121	
	N	8	8	8	8

**Correlation is significant for p<0,01 (twotailed).

*Correlation is significant for p<0,05 (twotailed).

**Table 3:** Correlations BMC+CPG group.

		CBCT	MDCT	Histomorphometrycortical	Histomorphometrycentral
CBCT	Pearson’s rank correlation (coefficient: r)	1	0.918**	0.922**	0.889**
	p - value		0.001	0.001	0.003
	N	8	8	8	8
MDCT	Pearson’s rank correlation (coefficient: r)	0.918**	1	0.817*	0.811*
	p - value	0.001		0.013	0.015
	N	8	8	8	8
Histmorphometry cortical	Pearson’s rank correlation (coefficient: r)	0.922**	0.817*	1	0.975**
	p - value	0.001	0.013		<0.001
	N	8	8	8	8
Histomorphometry central	Pearson’s rank correlation (coefficient: r)	0.889**	0.811*	0.975**	1
	p - value	0.003	0.015	<0.001	
	N	8	8	8	8

**Correlation is significant for p<0,01 (twotailed).

*Correlation is significant for p<0,05 (twotailed).

**Table 4:** Correlations BMC+CPG+PRP group.

		CBCT	MDCT	Histomorphometrycortical	Histomorphometrycentral
CBCT	Pearson’s rank correlation (coefficient: r)	1	0.963**	0.974**	0.901**
	p - value		<0.001	<0.001	0.002
	N	8	8	8	8
MDCT	Pearson’s rank correlation (coefficient: r)	0.963**	1	0.950**	0.785*
	p - value	<0.001		<0.001	0.021
	N	8	8	8	8
Histmorphometry cortical	Pearson’s rank correlation (coefficient: r)	0.974**	0.950**	1	0.873**
	p - value	<0.001	<0.001		0.005
	N	8	8	8	8
Histomorphometry central	Pearson’s rank correlation (coefficient: r)	0.901**	0.785*	0.873**	1
	p - value	0.002	0.021	0.005	
	N	8	8	8	8

**Correlation is significant for p<0,01 (twotailed).

*Correlation is significant for p<0,05 (twotailed).

## Discussion

Within the framework of this animal study, it could be demonstrated that the combined application of BMC+CPG+PRP had a positive influence on bone defect healing after six weeks compared to the application of BMC+CPG and the CPG alone. This is backed by the outcome of the quantitative MDCT- and CBCT-evaluation, but also by the analysis of the histomorphometrical results. Radiologically and histomorphometrically (central defect zone) a significantly higher bone regeneration could be determined in the BMC+CPG+PRP group. In the cortical defect zone the BMC+CPG+PRP group showed a higher new bone formation by trend, although the differences were not significant. When comparing the total composite of BMC+CPG+PRP to the autograft group the quantitative radiological and histomorphometrical evaluations showed no significant differences between these two groups. Thus, within the limitations of our study the application of the composite BMC+CPG+PRP represents an comparable alternative to autologous bone grafting during the early phase of bone healing after six weeks. In only very few histological slides of every treatment group an overgrowth of new bone above the cortical defect zone was observed. This overgrowing new bone formation was not quantified due to the rarity of this phenomenon and as it was located outside of the defect. This reaction was presumably part of the bone remodelling process in rare cases of this animal model. For these reasons the amount was not quantified.

In order to ensure the validity of the results of this study for human beings, mini-pigs were chosen as test animals. These animals possess a bone growth rate of 1.2–1.5 mm/per day, which is almost equal to the osseous reparative capacities of a human being [29].

The additional support of bone healing with the application of mesenchymal bone stem cells with additional use of PRP was also mentioned by other authors [11], [14]. Only a few studies evaluate the combination of bone marrow concentrate (BMC) with PRP being used on a weight-bearing long bone. Most studies focus on ex-vivo expanded mesenchymal stem cells and their interaction with PRP [30]. The ex-vivo expansion of mesenchymal stem cells, however, holds various problems, e.g. sterility of the cell culture, slow growth of the culture, which require a time-delayed second operation of the patient, as well as high costs [30]. An alternative approach could be the perioperative stem cell concentration by means of density gradient centrifugation of autologous bone marrow [14], [15]. Compared to autologous bone transplantations the donor site morbidity of the BMC procedure is reported to be lower [31]–[33].

Dallari et al. adopted bone marrow concentrate in combination with autologous PRP [34]. They conducted animal experiments on a metaphyseal critical size defect of a rabbit femur. For the therapy of the bone defect Dallari et al. used freeze-dried bone allograft alone or in combination with PRP and/or BMC [34]. At all time points of the experiment histomorphometrically the highest rate of new bone growth could be found within the BMC+allograft+PRP group. However, the authors did neither include a control group with autologous cancellous graft nor a radiological evaluation.

Furthermore, it should be critically discussed that no ceramic or biodegradable scaffold was used [34]. Especially the type and quality of the bone substitute seem to play an important role for the supportive effects of PRP on the bone healing process. In spite of many positive results the success of PRP, especially in the combination with synthetic bone substitutes, has been discussed controversially [3], [12], [35]. PRP does not always seem to have a positive effect on the bone healing process in combination with all bone substitutes or animal models [3]. With different synthetic bone substitutes the type and form of the material, the size of its pores (macro- or micropores) and particles, as well as the interconnectivity of the pores and its resorption rate seem to play an important role [3], [35], [36].

All these studies demonstrate that there is still no clear consensus regarding the “ideal” characteristics of artificial bone substitution materials for a combined use with PRP.

The successful impact of PRP in combination with micro- and macroporous calcium phosphate granules in our study may be explained by the specific material features of the granules. First of all, the granules are comprised of carbonated, calcium-deficient, apatitic CaP and exhibit a closer resemblance to human bone than stochiometric HA or TCP ceramics [37]. In addition, the interconnected network of micro- and macropores promote and allow bony ingrowth within the threedimensional geometry of the scaffold [38].

According to Bohner and Baumgart, the in vivo resorption rate of porous granules can be predicted to be much faster than in dense blocks or prismatic objects made out of the same material [39]. Furthermore, Tas et al. demonstrated that the same calcium phosphate granules, as we used in our study, were able to almost fully absorb fluid PRP prior to its implantation, which is based on a high wicking ability of the granules [37]. This may lead to a more extensive and longer absorption of the PRP by the granules, which may cause both continuous ingrowth from the outer periphery and throughout the entire scaffold.

The assignment of colony-forming units for fibroblastic cells (CFU-F) as a quality characteristic for mesenchymal stem cells goes back to the studies of Hernigou et al. [18]. CFU-F serve as an indicator for the activity of stromal cells and indirectly give a clue about the number of transplanted progenitor cells [40]. Other authors were furthermore able to prove that CFUs can be stained for osteogenic markers such as alkaline phosphatase (ALP) and that these cells will differentiate into osteogenic precursors [41]. For this reason we did not only determine the amount of CFU-F as a marker for fibroblastic cells, but also the amount of CFU-ALP in the aspirate and concentrate as a marker for osteoblastic progenitor cells. The ratio of CFU-ALP/CFU-F was 68% in BM and 56.5% in BMC indicating a differentiation into osteoblastic cells within the mononuclear cells of the BM and BMC. In this study the average amount of colony-forming units was 5.6 and 7.1 CFU-F/cm^2^ for BM and BMC, respectively, using a seeding density of 1×10^5^ WBC/cm^2^. For the CFU-ALP the values were 1.3 and 3.3 CFU-F/cm^2^ for BM and BMC. These values are comparable with the results of Jaeger et al. using human cells resulting in 2.8 CFU-F/cm^2^ for BM and 4.1 CFU-F/cm^2^ for BMC, respectively in a seeding density of 1×10^5^ WBC/cm^2^ [42]. Our values are also comparable to those published by Castro-Malaspina et al. [43] with 0.6–1.9 CFU-F/cm^2^ and Muschler et al. [44] with an average of 5.5 CFU/cm^2^ (seeding density of 1.2×10^5^ human WBC/cm^2^).

Within the framework of our study we were able to reach a significant increase in the concentration of the mononuclear cells within BMC by the factor 3.5 (p<0.006). However, our presented data displays a lower factor than Thoesen et al. who gained a 7-fold increase of nucleated cells in 19 adult dogs after the use of another BM concentration system [45]. Jager et al described a 5.2-fold enrichment in WBC in 37 patients with volumetric bone deficiencies [42] and Hermann et al. a concentration factor of 4.4 for total nuclear cells using both the same system [46]. Our lower factor might be due to the fact that the system used has only been developed for use of human BM. Any species related differences in cell density might tribute to a different concentration factor of BMC.

A very decisive parameter for the performance of PRP is implied in its production process, since this can influence the concentration of platelets and the concentration of the released growth factors significantly [47]. The concentration of platelets necessary for a positive PRP-effect on the bone regeneration seems to lie within a small range. For bone regeneration platelet concentrations that are 3 to 5 times the concentration of the plasma (about 1.000.000/µl) seem to be very advantageous according to Weibrich et al. and Marx et al. [6], [47], [48]. Below this concentration level the effect of PRP seems to be suboptimal and, paradoxically, higher concentrations have a repressing effect on the bone regeneration [47], [48]. With the method to produce PRP used in this study an average platelet count that is 4.7-fold higher compared to native blood was reached, whereby an optimal concentration for a possibly positive PRP-effect could be achieved. Another quality characteristic for PRP is implied in determining the concentration of growth factors contained in PRP [6], [49], [50]. In this context the studies of Lucarelli et al. are crucial, since their in-vitro experiments were able to prove that mesenchymal stem cells from bone marrow possess receptors for the growth factors contained in PRP [51]. Marx et al. assigned especially the growth factors PDGF and TGF-β an important role in bone healing [6], [50]. The significant concentration increase of the aforementioned growth factors with our method of PRP production could have had a decisive influence on the positive effect of PRP. With most animal experiments the studies lack quantitative determination of growth factors, which might be a possible explanation for the missing success [3].

The CT analysis represents a limitation of our study. Concerning absolute values, consolidation in CBCT as well as MDCT were essentially higher than in histomorphometry. This overestimation can be mainly explained by the fact that in both CT evaluations newly formed bone and remaining composites of CPG in the defect cannot be discriminated because of similar density. Therefore, according to Riegger and Kroepil et al., these two “tissues” were subsumed as bone consolidation [24], [26]. Additionally, methodical differences between CT evaluations measuring the entire defect and histomorphometry, which is performed on 3 single microscopic slices out of the defect have to be kept in mind. The highly significant correlation between histomorphometrical reference standard and measurements of CBCT as well as MDCT, however, indicates that both CT methods represent a reliable non invasive tool for monitoring of osseous consolidation in this animal model.

A further limitation of the validity of this study lies certainly in our time frame of six weeks. This time frame was chosen intentionally in order to evaluate the effects of PRP in the early phase of bone healing described by several authors [52], [53]. Within the limitations of this study, BMC+CPG in combination with PRP led to a comparable new bone formation in an in-vivo defect on load-bearing long bones of mini-pigs compared to the autograft group in the early phase of bone healing after six weeks. These positive effects of PRP in the early phase of bone healing shown in our study could possibly lead to a follow-up experiment with an extended time frame of possibly 12 weeks.

## Conclusions

Within the limitations of this study, the following statements about the treatment of metaphyseal critical-size defects on load bearing long bones of mini-pigs with autologous bone marrow concentrate (BMC) in combination with autologous PRP can be made:

BMC in combination with calcium phosphate granules (CPG) and autologous PRP led to a significantly higher new bone formation in the early phase of bone healing after six weeks as compared to the exclusive application of BMC combined with CPG. Besides, the composite BMC+CPG+PRP is comparable to autologous bone grafting within our experimental constellation. Further studies are needed to find out the effectiveness and transferability of this composite on diaphyseal defects.
